# CMR Assessment of endothelial damage and angiogenesis in porcine coronary arteries using gadofosveset

**DOI:** 10.1186/1532-429X-13-10

**Published:** 2011-01-26

**Authors:** Steen F Pedersen, Samuel A Thrysøe, William P Paaske, Troels Thim, Erling Falk, Steffen Ringgaard, Won Y Kim

**Affiliations:** 1 Dept. of Cardiothoracic and Vascular Surgery T, Aarhus University Hospital Skejby, Brendstrupsgaardsvej 100, 8200 Aarhus N, Denmark; 2 Dept. of Cardiology, Aarhus University Hospital Skejby, Brendstrupsgaardsvej 100, 8200 Aarhus N, Denmark; 3 MR-center, Aarhus University Hospital Skejby, Brendstrupsgaardsvej 100, 8200 Aarhus N, Denmark

## Abstract

**Background:**

Endothelial damage and angiogenesis are essential for atherosclerotic plaque development and destabilization. We sought to examine whether contrast enhanced cardiovascular magnetic resonance (CMR) using gadofosveset could show endothelial damage and neovessel formation in balloon injured porcine coronary arteries.

**Methods and Results:**

Data were obtained from seven pigs that all underwent balloon injury of the left anterior descending coronary artery (LAD) to induce endothelial damage and angiogenesis. Between one - 12 days (average four) after balloon injury, in vivo and ex vivo T1-weighted coronary CMR was performed after intravenous injection of gadofosveset. Post contrast, CMR showed contrast enhancement of the coronary arteries with a selective and time-dependent average expansion of the injured LAD segment area of 45% (p = 0.04; CI_95 _= [15%-75%]), indicating local extravasation of gadofosveset. Vascular and perivascular extravasation of albumin (marker of endothelial leakiness) and gadofosveset was demonstrated with agreement between Evans blue staining and ex vivo CMR contrast enhancement (p = 0.026). Coronary MRI contrast enhancement and local microvessel density determined by microscopic examination correlated (ρ = 0.82, p < 0.001).

**Conclusion:**

Contrast enhanced coronary CMR with gadofosveset can detect experimentally induced endothelial damage and angiogenesis in the porcine coronary artery wall.

## Introduction

Atherosclerotic plaque rupture and erosion is by far the most frequent cause of coronary thrombosis leading to myocardial infarction or sudden coronary death. Endothelial damage and angiogenesis are considered essential for atherosclerotic plaque development and destabilization [1-3]. Traditional X-ray coronary angiography is the clinical gold standard for diagnosing the presence and extent of coronary artery disease (CAD), but this technique is invasive and visualizes only the vessel lumen and not the plaque composition. For this reason, development of a non-invasive imaging technique to identify endothelial damage and angiogenesis in the coronary artery wall would have clinical implications.

Cardiovascular Magnetic Resonance (CMR) is a non-invasive imaging technique that has emerged as one of the most promising imaging modalities for assessing atherosclerosis in larger arteries [4-8]. However, detection of plaque components in the coronary arteries remains challenging due to respiratory and cardiac motion combined with the need for high spatial resolution. Therefore, CMR contrast agents that improve detection and characterization of coronary atherosclerotic plaques are needed.

Gadofosveset (Ablavar, Lanteus Medical Imaging, MA, USA) is a gadolinium based blood pool contrast agent used for magnetic resonance angiography [9]. It binds reversibly to albumin and is predominantly present within the vessel lumen but may enter the artery wall to a greater degree through the leaky microvessels or the damaged endothelium of atherosclerotic plaques [10,11].

Previous studies have shown that vulnerable plaque features, such as endothelial damage, angiogenesis, and inflammation can be induced in porcine coronary arteries by balloon inflation resulting in overstretching [12]. For the purpose of inducing an overstretch injury to model such plaque vulnerability features, balloon injury was applied to the left anterior descending coronary artery (LAD) of ten female Danish Land Race pigs. We sought to determine whether contrast enhanced CMR with gadofosveset could detect damaged endothelium and microvessels in the injured coronary artery.

## Methods

### Animal Protocol

**Figure 1:**
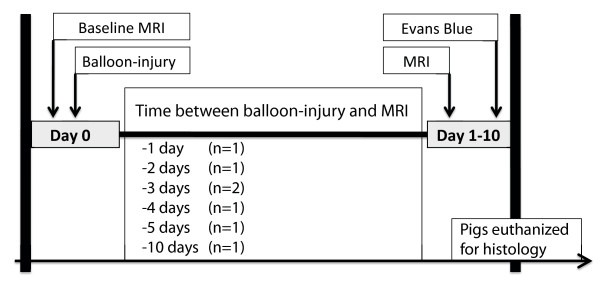
**Study design, showing the time course of the in vivo CMR experiment**. Balloon overstretch injury was induced in the LAD of 40 kg Danish Land Race pigs (n = 7). Post injury, gadofosveset enhanced coronary CMR was acquired. The animals were euthanized and ex vivo CMR observations were correlated with histopathological findings. The number of animals assessed by CMR at different time points after the balloon injury is indicated in parenthesis.

Ten female Danish Land Race pigs weighing 40 kg were used for the experiments (Figure 1). All pigs were treated according to the Danish law on animal experiments.

The pigs were pre-sedated with an intramuscular injection of stressnill (1 ml/kg) and midazolam (1 ml/kg). After induction of anesthesia with intravenous propofol (5 mg/kg) and endotracheal intubation, anesthesia was maintained with continuous intravenous infusion of propofol (8-10 mg/kg/hr) and fentanyl (2 mg/kg/hr). The pigs were mechanically ventilated with a tidal volume of 450 ml (respiratory rate 12/min).

The right common femoral artery was exposed by a surgical cut down, and a 9 F introducer sheath was inserted into the artery followed by a bolus injection of 100 IU/kg heparin administrated through the sheat. A 4.5 mm over the wire angioplasty balloon was placed in the LAD distal to the first diagonal branch. The coronary lesion was induced by two inflations at 12 atmospheres each lasting 30 seconds and with 60 seconds between the inflations.

Acetylsalicylic acid (100 mg/d) was given orally after the procedure and continued until euthanasia was induced by phenobarbital. All pigs underwent CMR followed by euthanasia between one and 12 days after the balloon injury depending on scanner availability and access to the animal laboratory.

### Contrast Agent

Gadofosveset trisodium is a stable gadolinium diethylenetriaminepentaacetic acid (Gd-DTPA) chelate derivative with a diphenylcyclohexylphosphate group; the molecular weight is 975.88 Da. It is applied as a small molecule blood-pool contrast agent. Depending on plasma concentration, 80% to 96% of gadofosveset is reversibly bound to human serum albumin. The binding to albumin sequesters most of the drug in the vascular space, yielding a distribution half-life of 46 min ± 11 and a fivefold increase in r1relaxation (~20-23 mM^-1^s^-1^) compared to non binding fraction of gadofosveset (~6 mM^-1^s^-1^) [13]. All animals received a standard dose of gadofosveset (0.03 mmol/kg) administered intravenously and followed by saline flush [10].

### In vivo CMR

CMR was performed on a 1.5T MR system (Intera, Philips Medical Systems, Best, The Netherlands) using a five element cardiac synergy coil. After a survey scan to localize the heart and diaphragm, a multi-heart phase steady state free precession (SSFP) cine sequence (repetition time (TR) 2.6 ms; echo time (TE) 1.3 ms; flip angle 60°; 50 heart phases; SENSE factor 2) was obtained to assess the interval of minimal LAD motion to determine the optimal trigger delay for the subsequent coronary scans.

First, a coronary non-contrast enhanced bright blood CMR angiography was performed with a previously described navigator-gated, free-breathing and cardiac-triggered T2 prepared 3D SSFP sequence allowing for the visualization of the anatomy of the coronary artery lumen (TR 4.9 ms; TE 2.5 ms; flip angle 90°) [14].

The contrast-enhanced coronary scan was a navigator-gated, free-breathing and cardiac-triggered T1-weighted inversion-recovery and fat-suppressed 3D SSFP sequence (TR 4.9 ms; TE 2.5 ms; flip angle 90°). Spatial resolution of both coronary scans was 1.3 × 1.3 × 3.0 mm^3 ^interpolated to 0.6 × 0.6 × 1.5 mm^3^. The contrast-enhanced coronary scan was performed once before contrast injection. After the intravenous administration of gadofosveset (0.3 mmol/kg), the scan was repeated every five minutes until the signal intensity from the coronary arteries had declined to a level that precluded the quantitative analysis.

The inversion time (TI) of the inversion recovery sequences was individually assessed before each acquisition according to the T1 of the myocardium using a TI scout (Look Locker sequence). Typically, the inversion time before contrast administration was 450-480 ms and after contrast application 300-325 ms.

### Ex vivo CMR

To document extravasation of contrast at the site of coronary injury, ex-vivo high resolution imaging was performed on the LAD in all pigs. The excised hearts were drained for blood and placed inside the same MRI scanner used for the in vivo imaging. A 47 mm circular micro-coil was applied over the LAD. After initial survey scans to locate the LAD, a high resolution IR 2D fast spin echo scan was performed (echo train length 8; TR 1000 ms; TE 20 ms; field of view (FOV) 4 × 4 cm; matrix 256 × 256; slice thickness 1.5 mm). A fat saturation pulse was used to eliminate the epicardial fat signal and thus enhance the definition of the outer boundary of the vessel. The inplane resolution obtained was 0.15 × 0.15 mm.

### Histopathology

After injection, practically all Evans blue binds to albumin and as a result only enters the vessel wall and perivascular area if there is a defect endothelial barrier [15-17]. In this study, the dye was used to ease the localization of the balloon-injured LAD segment and to macroscopically confirm that the balloon injury had caused increased vessel wall permeability. The solution was prepared by dissolving 2 g of Evans blue (Serva, Heidelberg, Germany) in 50 ml of isotonic saline. Evans blue was administered through an ear vein cannula and allowed to circulate in the bloodstream for 1 hr after which the pigs were killed and the hearts were harvested. After ex vivo CMR, the LAD was removed from the heart and cut into 4 mm consecutive cross sectional slices and subsequently photographed with a digital camera (Nikon, Tokyo, Japan) for the purpose of registering any uptake of Evans blue. Surrounding epicardial fat and myocardium were included in the section for arterial support during fixation and to improve matching with CMR through the use of anatomical markers. All LAD slices were fixated in formaldehyde and embedded in paraffin. Four micron thick sections were cut and processed for microscopic examination, including hematoxylin-eosin (H&E) and immunohistochemistry for angiogenesis (von Willebrand factor positive microvessels).

## Data analysis

### In vivo CMR

To assess extravasation of gadofosveset into the coronary artery wall and perivascular area, the 3D coronary data set was reformatted along the entire visualized course of the coronaries [18].

The balloon-injured area of the LAD was located visually by its relation to anatomical landmarks, such as side branches identified on both x-ray and CMR angiograms. The average areas of the LAD and circumflex artery were defined by manual tracing of the outer wall boundary on the reformatted images by two observers (SFP, ST) using the free software Osirix (version 2.5.7, Geneva, Switzerland). An increase in the vessel area over time was considered to reflect extravasation of contrast agent into the vessel wall and perivascular area.

### Ex vivo CMR

Arterial lumen and outer wall boundary were manually defined on each cross sectional CMR images using ImageJ software (National Institutes of Health) by one observer (SFP). A region of interest (ROI) was placed in a homogeneous region of the myocardium and the relative mean signal intensity (SI) was calculated as: SI_vessel_/SI_myocardium_. The balloon-injured (n = 7) and uninjured (n = 7) segments were scanned using multiple cross-sections per segment. If one or more of the CMR cross-sections were positive for enhancement, the entire segment was classified as enhanced. Positive CMR enhancement was defined as areas with SI 5 SD above the adjacent myocardium.

### Histopathology

One observer (SFP) reviewed all histopathology.

### Evans blue

Cross-sectional slices were prepared from both the balloon-injured and uninjured segments. Photos of the cross sectional sliced LAD segments were inspected and based on the presence or absence of an intense blue color in the vessel wall and perivascular area that was visually distinct from the surrounding tissue, each cross-section was categorized as being either positive or negative for Evans blue uptake. If one or more of the cross-sections contained Evans blue, the entire balloon-injured (n = 7) and uninjured (n = 7) segments were classified as Evans blue positive.

### Balloon injury

The H&E-stained sections from each balloon injured LAD segment were studied microscopically to determine if the balloon inflations had caused overstretch injury to the artery wall, defined as a total disconnection of the tunica media.

### Microvessel

To evaluate the microvessel density, the adventitia of interest was manually defined on the histological sections stained for von Willebrand factor using ImageJ software (National Institutes of Health). The microvessel density was calculated as follows:

Microvessels/mm^2 ^= Number of microvessels/vessel wall and adjacent adventitia area (mm^2^)

The average microvessel density was calculated for uninjured vs injured segments, respectively, and the results were compared.

### Ex vivo CMR versus Evans blue and Microvessels

Ex vivo CMR images were matched with the corresponding photos (Evans blue) and histological sections (microvessels) by using landmarks such as the relative distance from the left coronary ostium and gross morphological features such as the overall shape and size of the lumen and vessel wall.

The agreement between the occurrences of vessel wall enhancement on T1 weighted CMR images and Evans blue (endothelial leakiness) was determined by registering all matches and mismatches between the two variables. The average relative SI of the balloon injured and uninjured segments were calculated and compared. Finally, the relative SI of the balloon injured and uninjured segments were correlated to the average microvessel density measured on the corresponding histological slices.

### Statistical Analysis

The post-contrast increase in vessel area of the LAD and circumflex artery was tested by the signed Wilcoxon rank sum test. The association between gadofosveset enhancement of the vessel wall and uptake of Evans blue was tested with a 2-tailed Fischer's exact test. The average microvessel density and the relative CMR signal intensity for the balloon-injured and uninjured segments were compared, using a Wilcoxon signed rank test. The correlation between coronary vessel wall SI by CMR and microvessel density was investigated using Spearman correlation test. The interobserver and intraobserver variability were assessed by a 1-way random, 1-measure intraclass correlation coefficient (ICC). The statistical analysis was performed using SPSS software version 18.0 (SPSS Inc. Chicago, IL).

## Results

Ten pigs were subjected to the experimental protocol, three of the pigs died of ventricular fibrillation during the balloon intervention leaving seven pigs for further investigation. All in and ex vivo CMR examinations of the remaining pigs were successfully performed.

### In vivo CMR

The non contrast enhanced coronary angiograms did not show any visual signs of injury to the LAD. Twenty minutes post gadofosveset injection, suboptimal image quality caused by tachycardia necessitated the exclusion of two of the pigs. Thus, five of the pigs have data for the full length of observation (60 minutes).

**Figure 2:**
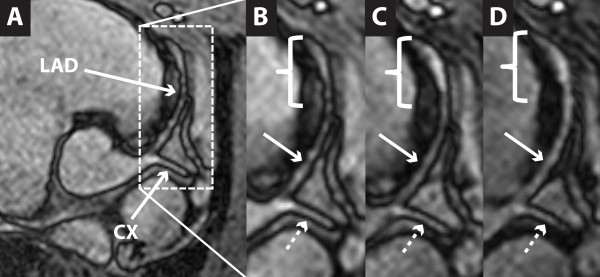
**Coronary bright-blood CMR-angiography** (A). T1-weighted inversion recovery at 5 min (B) 15 min (C), and 25 min (D) following intravenous administration of gadofosveset. The area of the balloon injured LAD2 segment expands over time indicating time-dependent extravasation of contrast, whereas the intact LAD1 segment (arrow) and CX (dotted arrow) remain constant.

**Figure 3:**
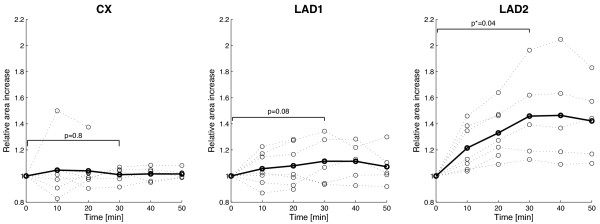
**Expansion of the contrast enhanced vessel area (CX, LAD1, and injured LAD2) as a function of time for all pigs (dotted lines) and average (bold lines). **Thirty minutes post contrast, the injured LAD2 segment showed an average expansion of 45% (p = 0.04) while the average area of the uninjured CX and LAD1 artery remained constant with an average expansion of 7% (p = 0.8) and 11% (p = 0.08), respectively. In two pigs, images obtained 20 minutes post injection of gadofosveset were excluded due to poor image quality.

The gadofosveset enhanced angiograms showed a selective and time-dependent expansion of the injured LAD segment area until 30 minutes after the administration of gadofosveset after which it reached a plateau (Figure 2 and 3). Thirty minutes post contrast, CMR of the injured LAD segment demonstrated contrast enhancement of the vessel yielding a significant increase in the average artery area of 45% (p = 0.04; CI_95_= [15%-75%]) while the average area of the uninjured circumflex artery (CX) and uninjured part of the LAD artery remained constant with an average increase of 7% (p **= **0.8; [1%-12%]) and 11% (p = 0.08; [-6%-27%]), respectively. Using a cut-off-value of 10% relative area increase, we found both the sensitivity and specificity to be 100% at thirty minutes post contrast.

## Histopathology

### Evans blue

A total of sixty-five LAD cross sections were photographed for the purpose of documenting up-take of Evans blue into the vessel wall. Five of these photos were excluded due to insufficient image quality and additional 10 photos were excluded due to non-successful matching to the corresponding ex vivo CMR leaving 50 cross-sectional photos for further investigation (average seven photos pr pig). In the seven balloon-injured segments, a total of thirty cross-sections were obtained. For the seven uninjured segments, 20 cross-sections were obtained. Based on the photo observations, 32 cross-sections were classified as being Evans blue positive, with 27 (84%) originating from balloon-injured segments. Eighteen cross-sections were classified as being negative for Evans blue uptake, with 15 (83%) originating from uninjured cross-sections.

### Balloon injury

The LAD segments that were exposed to balloon overstretch all displayed ruptured tunica media, i.e., total disconnection of the tunica media, on the H&E stained histological sections, indicating balloon injury.

### Microvessels

Sixty-five histological sections were immunohistochemically stained for von Willebrand factor. The histological sections could be matched with the ex vivo CMR images in 30 cases (average four pr pig). The difference in average microvessel density between the balloon injured and uninjured segments was 7.6 vessel/mm^2 ^(p = 0.075).

### Ex-vivo CMR

On average, the balloon injured segments were 34% more enhanced than the uninjured segments (p = 0.043).

### Ex vivo CMR versus histopathology

**Figure 4:**
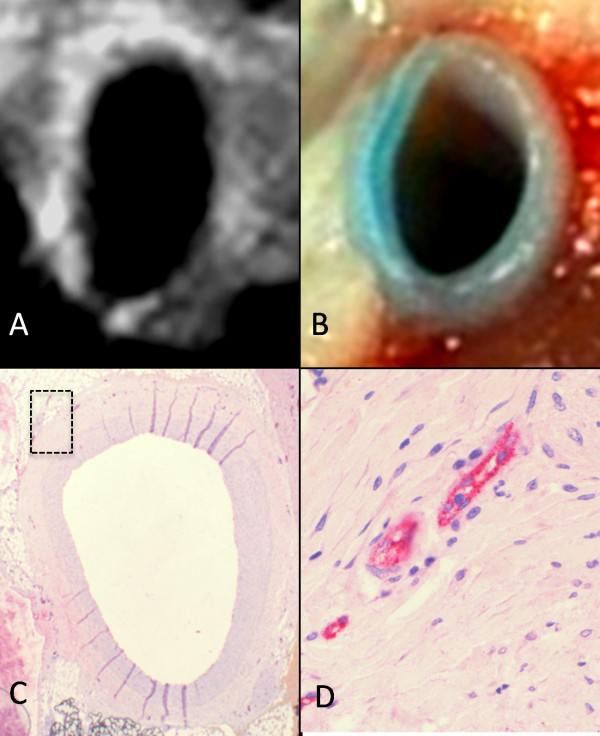
**Ex vivo T1-weighted CMR images of a balloon injured porcine coronary artery after injection of gadofosveset **(A). Photo of the corresponding segment demonstrating extravasation of Evans blue into the arterial wall (B). Histological identification of microvessels (positive for von Willebrand factor). Note the strong CMR signal located at the coronary artery wall after injection of gadofosveset corresponding to the area with uptake of Evans blue and to microvessel-rich areas in the histological section (C+D).

**Figure 5:**
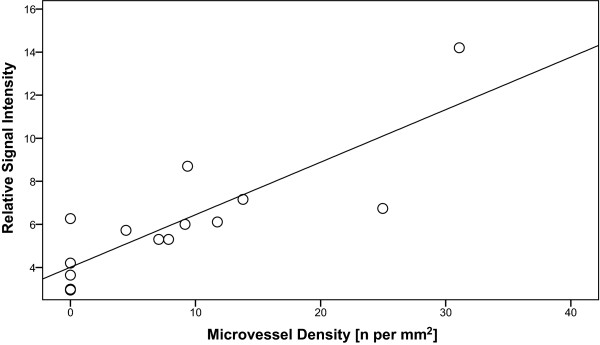
**Mean microvessel density determined by histology (number per mm^2^) was plotted against mean relative CMR signal intensity in both injured and uninjured LAD vessel wall segments**. The CMR signal intensity of the vessel wall was calculated relative to the adjacent myocardium. The regression line indicates the relationship between the two variables (ρ = 0.82, p < 0.001).

Gadofosveset contrast enhancement of the injured arterial wall was documented by ex vivo CMR. We detected enhancement in 24 out of 32 Evans blue positive cross sections and in 1 out of 18 Evans blue negative cross sections. There was a significant association between segments showing positive coronary artery wall enhancement and segments showing Evans blue uptake (p = 0.026) (Table 1 and Figure 4). There was a significant correlation between the average SI of the LAD segments and average segment microvessel density (Spearman's ρ = 0.82, p < 0.001) (Figure 5).

**Table 1:** Correlation between Evans blue and MR enhancement

	Evans Blue: +	Evans Blue: -	Total
**MR Enhancement: +**	8	0	8
**MR Enhancement: -**	2	4	6
**Total**	10	4	14

Observed agreement of CMR enhancement and Evans blue uptake calculated for both injured and uninjured segments. Positive CMR enhancement was defined as areas with SI 5 SD above the adjacent myocardium and Evans blue was defined as an intense blue color in the vessel wall and perivascular area that was visually distinct from the surrounding tissue. (+) = Yes (-) = No

### Interobserver and intraobserver agreement

Interobserver and intraobserver agreement for the assessment of in vivo coronary vessel areas were 0.94 (p < 0.001) CI_95 _= [0.92 - 0.97]) and 0.95 (p < 0.001) CI_95 _= [0.94- 0.97], respectively.

## Discussion

We have demonstrated that CMR in combination with gadofosveset, an intravascular contrast agent with a high affinity to albumin, enables in vivo differentiation between injured and normal porcine coronary artery walls. In addition, we have shown that the contrast enhancement of the coronary artery wall was in good agreement with endothelial damage assessed by Evans blue and correlated with microvessel density measured on corresponding arterial sections with immunohistochemistry.

We detected contrast enhancement in 24 of 32 (75%) injured coronary cross sections while enhancement occurred in only one out of 18 (6%) uninjured coronary cross sections. The most likely mechanism is that albumin enters the injured lesions through damaged or missing endothelium and leaky microvessels [10,19]. Since 80% of gadofosveset will be bound to serum albumin, it will extravasate into the injured artery wall together with albumin. In addition, unbound extracellular gadofosveset may also bind directly to lesion resident albumin in the artery wall. This observation is consistent with observations by Kooi et al. who recently described that gadofosveset showed specific signal enhancement in the artery wall of atherosclerotic rabbits containing an increased number of microvessels, whereas only limited enhancement was seen in normal vessels [10]. Due to the gradual uptake of gadofosveset into the injured coronary vessel wall, the optimal time for combined lumen and vessel wall imaging would be approximately 30 minutes after contrast injection. The relative area increase of the proximal uninjured LAD segment appeared greater than in the uninjured CX segment indicating that the proximal LAD segment was injured in some cases, which was confirmed by the detection of Evans blue in 5 (25%) out of the 20 uninjured cross sectional LAD slices. The unintended injuries to the proximal LAD most likely occurred doing the withdrawal of the balloon causing denudation of the endothelium.

One of the pigs was imaged at day one after the balloon injury, which is too early for neovessels to develop in the lesion area. Despite the lack of neovessels, the diameter of the vessel still increased, indicating that endothelial damage alone can cause extravasation of gadofosveset into the vessel wall. Consequently, we were unable to discriminate between endothelial damage and neovessels.

### Previous approaches for imaging of atherosclerotic plaque

Low molecular weight gadolinium chelates, like Gd-DTPA (gadopentetatedimeglumine), has been used to improve atherosclerotic plaque detection [5,20-23]. One major limitation for low molecular contrast agents is that these small molecular weight probes are known to diffuse quickly from the blood into the extracellular space and enters both the normal and atherosclerotic vessel walls [10]. The differentiation between stable and vulnerable plaques using Gd-DTPA and delayed enhancement CMR is therefore difficult. This is reflected in patient studies where enhancement of coronary plaques has been observed both in patients with stable angina and in patients with acute coronary syndromes [24,25]. Dynamic contrast enhanced CMR combined with pharmacokinetic analysis of Gd-DTPA uptake has been shown to correlate with microvessel density in human carotid plaques [26], but due to cardiac motion this approach is challenging for the coronary arteries.

Recently, other gadolinium based contrast agents such as Gadoflurine and B-22956/1 (Gadocoletic acid trisodiumsalt) have been shown to enhance specific components associated with advanced atherosclerotic plaques in atherosclerotic rabbits [27,28]. These contrast agents could potentially be used for atherosclerotic plaque detection in the future.

Vulnerable plaque imaging can also be performed using ultra small super paramagnetic iron oxide (USPIO) particles, which enables detection of macrophages in atherosclerotic plaques of atherosclerotic rabbits and human carotid arteries [29,30]. After phagocytosis by macrophages, USPIO can be detected through susceptibility effects using T2* weighted CMR sequences. However, to our knowledge, this has never been demonstrated in the coronary arteries and since the best imaging time point after injection of USPIO is reported to be around 36 hours, the clinical use may be limited.

### Limitations

In this study, we demonstrated that mechanically induced endothelial damage and angiogenesis could be visualized using Gadofosveset. Although endothelial damage and angiogenesis are features of advanced atherosclerotic plaques, other vulnerable plaque components are missing such as a lipid-rich necrotic core with a fibrous cap. Validation in animal models with atherosclerosis is warranted before judging the applicability of the method for assessing advanced human plaques.

Matching the ex vivo CMR to histology is challenging and associated with a certain level of inaccuracy despite careful matching, given the large disparity in slice thickness (4 μm for histology and 1.5 mm for CMR).

## Conclusion

The contrast agent gadofosveset allowed for non-invasive detection of endothelial damage and angiogenesis in balloon injured porcine coronary arteries. Therefore, gadofosveset enhanced CMR has a potential to identify vulnerable atherosclerotic plaques in human coronary arteries.

## Competing interests

The authors declare that they have no competing interests.

## Authors' contributions

SFP carried out all the procedures related to the animal model performed all the CMR scans, analyzed the acquired CMR and histological data, contributed to the statistical analyses, obtained all illustrations, wrote the manuscript, and merged all feedback from the co-authors into the final manuscript. ST contributed to the analysis of the acquired CMR and histological data, the statistical analyses, the illustrations and drafting the manuscript and revised it critically for intellectual content. WP took part in formulating the study, revised the manuscript critically and contributed to important intellectual content of the manuscript. TT contributed to procedures related to the animal model, and drafting the manuscript and revised it critically for intellectual content. EF contributed to the study design, drafting the manuscript, and revised it critically, for intellectual content. SR contributed to the CMR sequence setup, revised the manuscript critically and contributed to important intellectual content of the manuscript. WYK contributed to the study design, the CMR sequence setup, drafting the manuscript and revised it critically for intellectual content. All authors have read and approved the final manuscript.
